# DNA methylation and behavioral dysfunction in males with 47,XXY and 49,XXXXY: a pilot study

**DOI:** 10.1186/s13148-021-01123-4

**Published:** 2021-07-01

**Authors:** Richard S. Lee, Sophia Q. Song, Henri M. Garrison-Desany, Jenny L. Carey, Patricia Lasutschinkow, Andrew Zabel, Joseph Bressler, Andrea Gropman, Carole Samango-Sprouse

**Affiliations:** 1 The Mood Disorders Center, Department of Psychiatry and Behavioral Sciences, Johns Hopkins University School of Medicine, Baltimore, MD USA; 2 Department of Research, The Focus Foundation, Davidsonville, MD USA; 3 Department of Epidemiology, Johns Hopkins University Bloomberg School of Public Health, Baltimore, MD USA; 4 Kennedy Krieger Institute, Baltimore, MD USA; 5 Department of Neurology, George Washington University, Washington, DC USA; 6 Department of Pediatrics, George Washington University, Washington, DC USA; 7 Division of Neurogenetics and Developmental Pediatrics, Children’s National Health System, Washington, DC USA; 8 Department of Human and Molecular Genetics, Florida International University, Miami, FL USA

**Keywords:** Sex chromosome aneuploidies, DNA methylation, MAOA, 47,XXY, 49,XXXXY, Executive functioning, Externalizing disorders, Internalizing disorders

## Abstract

**Background:**

Equal dosage of X-linked genes between males and females is maintained by the X-inactivation of the second X chromosome in females through epigenetic mechanisms. Boys with aneuploidy of the X chromosome exhibit a host of symptoms such as low fertility, musculoskeletal anomalies, and cognitive and behavioral deficits that are presumed to be caused by the abnormal dosage of these genes. The objective of this pilot study is to assess the relationship between CpG methylation, an epigenetic modification, at several genes on the X chromosome and behavioral dysfunction in boys with supernumerary X chromosomes.

**Results:**

Two parental questionnaires, the Behavior Rating Inventory of Executive Function (BRIEF) and Child Behavior Checklist (CBCL), were analyzed, and they showed expected differences in both internal and external behaviors between neurotypical (46,XY) boys and boys with 49,XXXXY. There were several CpGs in *AR* and *MAOA* of boys with 49,XXXXY whose methylation levels were skewed from levels predicted from having one active (Xa) and three inactive (Xi) X chromosomes. Further, methylation levels of multiple CpGs in *MAOA* showed nominally significant association with externalizing behavior on the CBCL, and the methylation level of one CpG in *AR* showed nominally significant association with the BRIEF Regulation Index.

**Conclusions:**

Boys with 49,XXXXY displayed higher levels of CpG methylation at regulatory intronic regions in X-linked genes encoding the androgen receptor (*AR*) and monoamine oxidase A (*MAOA*), compared to that in boys with 47,XXY and neurotypical boys. Our pilot study results suggest a link between CpG methylation levels and behavior in boys with 49,XXXXY.

**Supplementary Information:**

The online version contains supplementary material available at 10.1186/s13148-021-01123-4.

## Background

47,XXY (Klinefelter syndrome or KS) is an X and Y chromosomal variation that results from the addition of an extra X chromosome, affecting 1 out of every 660 live male births [1–3]. 49,XXXXY is a more severe variant of 47,XXY that occurs in 1 out of every 85,000 to 100,000 live male births [4–6]. Both sex chromosome aneuploidies (SCA) commonly exhibit testicular dysgenesis and hypergonadotropic hypogonadism attributable to the one or more additional X chromosomes, and the decreased levels of testosterone in these males also may affect neurocognitive development [7–10]. Behaviorally, patients with 47,XXY may have elevated levels of anxiety, peer social interactional differences, and internalizing problems when compared to neurotypical standards, according to the Child Behavior Checklist (CBCL) [11]. Boys with 49,XXXXY have exhibited elevated scores on similar domains of the CBCL, including anxiety, internalizing problems, thought problems, and externalizing problems to an increased extent than as described for 47,XXY [11].

Previous studies suggest the benefit of hormonal replacement therapy (HRT) on neurocognitive, neurobehavioral, and neuromotor capabilities for boys with 47,XXY and 49,XXXXY. Pediatric endocrinologists currently prescribe HRT, specifically testosterone, to males with 47,XXY and 49,XXXXY based on physical examination. Neurodevelopmental investigations on these males showed that infants with 47,XXY who receive early hormonal treatment (EHT) showed improvement in Full-Scale IQ, Verbal IQ, and speech and language development when compared to untreated boys [12]. In addition, parents of treated boys reported significantly fewer behavioral concerns along with improved social behavioral and initiation skills [13]. Males with 47,XXY who were treated with HRT experienced fewer social, thought, and affective problems when compared to the untreated group [14]. In a recent study, infants with 49,XXXXY who received EHT scored higher on the Bayley Scales of Infant Development compared to their untreated counterparts [7]. While such studies suggest global improvement in these populations with testosterone treatment, the etiology of these behaviors has not been investigated from a genetic perspective [13, 15].

It has been hypothesized that the behavioral variation between these patients may be attributed to the presence and skewed inactivation of their additional X chromosomes. In the neurotypical 46,XX female, one of the two X chromosomes is inactivated in order to silence the transcriptional activity of the second sex chromosome. This X inactivation (Xi) allows the female to maintain an appropriately equal expression of sex-linked genes compared to her neurotypical 46,XY male counterpart. Inactivation is a complex process that depends on genes on autosomes that are activated by the presence of two X chromosomes and involves, in part, the transcription of the *XIST* (X-inactive specific transcript) RNA from the inactive X chromosome [16]. The inactive X chromosome is also associated with specific DNA methylation patterns that play a key role in the silencing of its genes [17, 18]. DNA methylation regulates gene expression by recruiting proteins involved in gene repression or by inhibiting the binding of transcription factors to DNA [19]. DNA methylation is therefore a powerful mechanism for the regulation of gene transcription.

When there are more than two X chromosomes, such as in 49,XXXXY syndrome, the typical pattern of DNA methylation might be skewed on the inactive X chromosome. Such aberrant X chromosome methylation patterns are likely to impact gene expression, possibly resulting in specific clinical phenotypes [20]. For instance, fragile X syndrome is caused by CGG expansion and hypermethylation at the *FMR1* (fragile X mental retardation 1) gene promoter, resulting in transcriptional repression [21]. The FMRP protein encoded by *FMR1* helps regulate synaptic plasticity, which is the hallmark of learning and memory. As such, fragile X syndrome is characterized by severe intellectual disability, autism spectrum disorder (ASD), language delay, sensory hyperarousal and anxiety [22]. While fragile X syndrome exemplifies the effects of hypermethylation of genes on the X chromosome, hypomethylation of genes on additive X chromosomes could also have potentially powerful phenotypic effects by disrupting the typical dosage of transcriptional activity.

This pilot study is the first to explore DNA methylation on genes on the X chromosome associated with behavior in patients with 47,XXY and 49,XXXXY and the possible interaction between HRT and DNA methylation levels. The behavior of patients with 47,XXY and 49,XXXXY is assessed using the BRIEF (Behavior Rating Inventory of Executive Function) and CBCL (Child Behavioral Checklist). These behavioral assessment results are then compared to epigenetic analyses of degree of DNA methylation at several X-linked genes. Our approach has the potential to elucidate the complex interaction between gene and behavior in individuals with multiple X chromosomes.

## Results

### BRIEF analysis

Detailed information on the participants, such as cohort size, diagnosis, and exclusion criteria, is provided in Materials and methods (Sect. 5.1). For the first analysis, individuals with 47,XXY were compared to individuals with 49,XXXXY using the BRIEF, which is a questionnaire administered to the participants’ parents and includes three scales to measure behavioral regulation, five scales for metacognition, and a composite score that combines all scales. The mean age of the twenty-six participants with 47,XXY (mean = 159.35 months; *SD* = 42.22) for whom BRIEF was available was significantly higher than the group of eleven participants with 49,XXXXY (mean = 102.27 months, *SD* = 26.10, *P* = 0.001). To control for this age disparity between groups, a subgroup of the youngest participants with 47,XXY (mean age = 119.18 months, *SD* = 25.01) was created. The mean age of this group of eleven younger participants with 47,XXY was not significantly different than the group of eleven participants with 49,XXXXY (*P* = 0.14). In the group of eleven younger participants with 47,XXY, none of the mean averages for the BRIEF Composite scales (i.e., Behavioral Regulation Index, Metacognition Index, and Global Executive Composite) were significantly different from a test value of *T* = 50 (*T* = 52.5 to 53.4). In contrast, mean averages for all of the BRIEF Composite scales were significantly higher than the test value of *T* = 50 in the 49,XXXXY group (*T* = 62.0 to 66.6, *P* < 0.01 for all comparisons using the Student’s *T*-test), suggesting a significantly higher level of parent-reported executive dysfunction in these individuals. Moreover, when the 47,XXY and 49,XXXXY groups were compared, significant differences were identified on all BRIEF categories (Table 1).

**Table 1:** Standardized measures (mean, *SD*) of behavior rating inventory of executive functions (BRIEF)

Test	Mean 47,XXY (*SD*)	Mean 49,XXXXY (*SD*)	Significance	Cohen's *d*
BRIEF Behavioral Regulation Index (*T*)	52.5 (15.2)	66.6 (13.9)	0.034	0.97
BRIEF Metacognition Index (*T*)	52.5 (12.6)	62.0 (8.0)	0.049	0.90
BRIEF Global Executive Composite (*T*)	53.4 (14.0)	64.7 (9.4)	0.037	0.95

*T* = *T*-score; *SD* = standard deviation; mean scores for the 49,XXXXY group were all significantly different from the test value *T* = 50 (*P* < 0.01)

### CBCL analysis

For the second analysis, 47,XXY and 49,XXXXY groups were compared using the CBCL, which is a questionnaire administered to the participants’ parents to measure the expression of internalizing problems such as anxiety and depression, externalizing problems, and other social problems. Once again, the mean age of the twenty-nine participants with 47,XXY (mean = 146.68 months; *SD* = 42.53) with available CBCL data was significantly higher (*P* = 0.003) than that of the group of thirteen participants with 49,XXXXY (mean = 104.94 months, *SD* = 29.06). To control for this age disparity between groups, a subgroup of the thirteen youngest participants with 47,XXY (mean age = 108.08 months; *SD* = 26.74) was created so that the mean age difference between the two groups was not significant (*P* = 0.78). In the thirteen individuals with 47,XXY, mean values for the CBCL Internalizing and Externalizing Problems scales (*T* = 55.7 and 53.9, respectively) were not elevated when compared to a reference value of *T* = 50, but the mean value for the Total Problems scale was significantly elevated (*T* = 58.9, *P* = 0.02). In contrast, mean values for the 49,XXXXY group were significantly higher than the test value of *T* = 50 for all three CBCL composite scales (*T* = 64.7 to 68.8, *P* < 0.001). Moreover, mean values for the two groups were significantly different for each of the composite scales on the CBCL, indicating significantly higher parent reports of internalizing, externalizing, and total behavioral problems in the 49,XXXXY group (Table 2).

**Table 2:** Standardized measures (mean, *SD*) of child behavior checklist (CBCL)

Test	Mean 47,XXY (*SD*)	Mean 49,XXXXY (*SD*)	Significance	Cohen's *d*
CBCL Internalizing Problems (*T*)	55.7 (13.1)	66.2 (10.4)	0.034	0.89
CBCL Externalizing Problems (*T*)	53.92 (9.6)	64.7 (8.6)	0.006	1.18
CBCL Total Problems (*T*)	58.9 (11.8)	68.8 (8.2)	0.020	0.97

*T* = *T*-score; *SD* = standard deviation; mean scores for the 49,XXXXY group were all significantly different from the test value *T* = 50 (*P* < 0.001)

### DNA methylation analysis

**Fig. 1:**
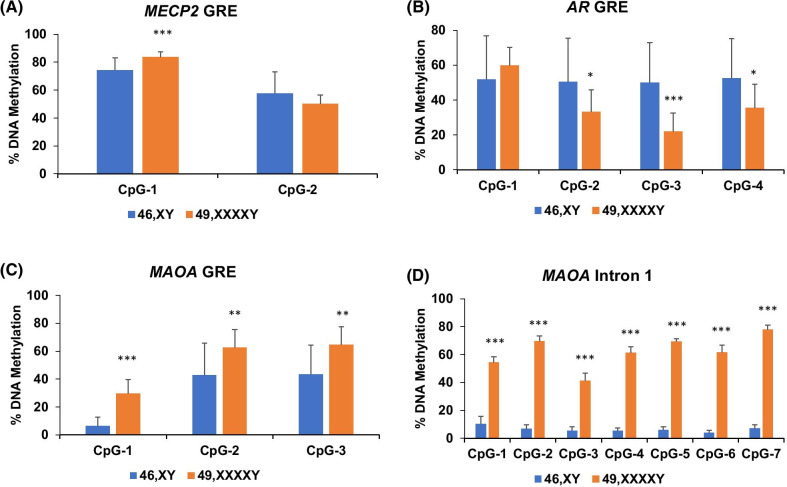
Methylation levels at candidate X-linked loci between 46,XY and 49,XXXXY. Genomic DNA extracted from saliva were used for bisulfite pyrosequencing of putative regulatory regions at three genes involved in neurodevelopment and neurotransmission: methyl CpG-binding protein 2 (*MeCP2*) (**a**), androgen receptor (*AR*) (**b**), and the third (**c**) and first (D) introns of monoamine oxidase A (*MAOA*). Error bars are presented as mean ± STD. *P < 0.05; **P < 0.01; and ***P < 0.001

To identify potential mechanisms that underlie the behavioral deficits in the individuals with 49,XXXXY, bisulfite pyrosequencing assays were conducted to compare saliva DNA between the 49,XXXXY and neurotypical 46,XY groups for methylation differences in a small selection of genes on the X chromosome. Since we had no a priori knowledge of specific CpG targets that have been implicated for individuals with 49,XXXXY, we focused on several X-linked candidate genes that were involved in neurodevelopment and behavior. Further, we chose CpGs adjacent to glucocorticoid response elements (GREs) given the role of glucocorticoids in neurodevelopment and behavior. Only one significant but subtle difference was observed at one of the two neighboring CpGs in the methyl-CpG binding protein 2 (*MeCP2)* gene (*P* = 3.5 × 10^−4^, Fig. 1a). At a GRE in the first intron of the androgen receptor (*AR*), significant decreases in DNA methylation were observed in three of the four CpG examined, although the standard deviations were high in both groups (*P* < 0.02, Fig. 1b). Interestingly, significant increase in DNA methylation was observed at all three CpGs at an intronic GRE in the monoamine oxidase A gene (*MAOA*) (*P* < 0.003, Fig. 1c). We also targeted a region adjacent to a CpG island in the first intron of *MAOA*, since its CpG methylation has been associated with MAOA enzymatic activity in the brain [30] and thus may play a role in the transcriptional regulation of *MAOA*. Six–eightfold increases in methylation were observed at each of the seven CpGs examined between the 49,XXXXY and 46,XY groups (*P* < 0.001, Fig. 1d).

### X chromosome methylation versus copy number analysis

**Fig. 2:**
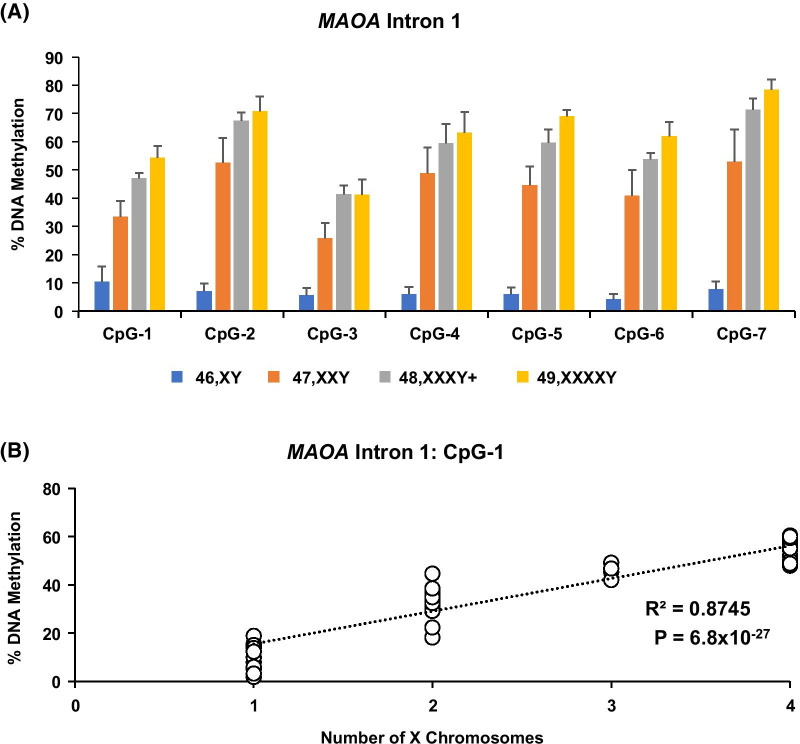
X chromosome number-dependent increase in MAOA methylation levels in 46,XY; 47,XXY; 48,XXXY+; and 49,XXXXY individuals. Bisulfite pyrosequencing of saliva DNA shows a dose-dependent increase in DNA methylation in intron 1 of the *MAOA* gene (**a**). Linear regression analysis for CpG-1 showed a strong correlation between observed DNA methylation and X chromosome number (**b**). The 48,XXXY + group consists of two individuals with 48,XXXY and one with 49,XXXYY karyotype. Groups with different numbers of X chromosomes (1–4) are: 46,XY; 47,XXY; 48,XXXY + ; and 49,XXXXY, respectively. Error bars are presented as mean ± STD

**Fig. 3:**
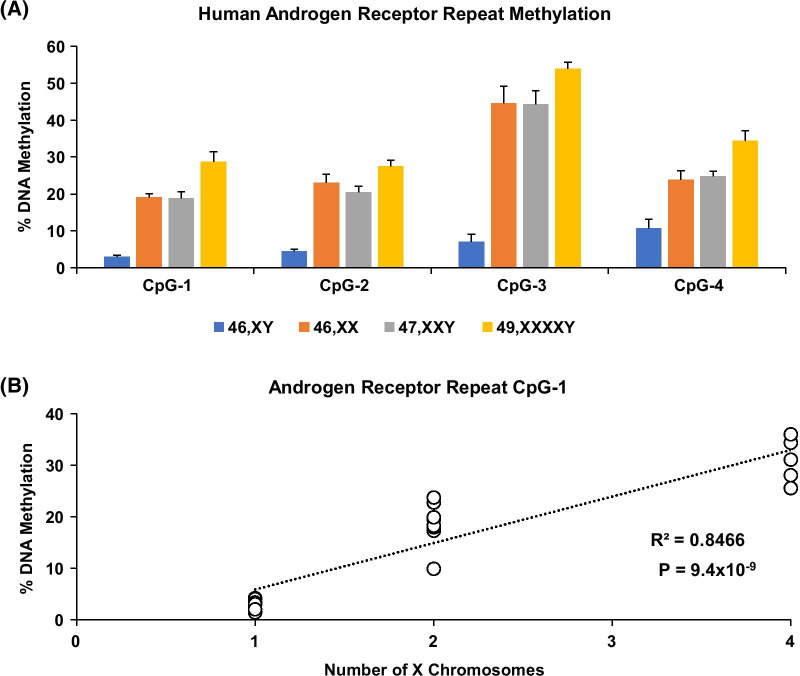
X chromosome number-dependent increase in AR methylation levels in 47,XXY and 49,XXXXY probands and their 46,XY or 46,XX parents and siblings. Bisulfite pyrosequencing of saliva DNA shows a dose-dependent increase in DNA methylation in exon 1 CAG tandem repeat of the AR gene (**a**). Linear regression analysis showed a strong correlation between observed DNA methylation levels and X chromosome number. Graph shows the regression results for CpG-1 (**b**). Note that the 46,XX and 47,XXY show the same methylation levels, suggesting that the second X chromosome in the XXY probands are inactivated. Groups with different numbers of X chromosomes (1–4) are: 46,XY; 46,XX; 47,XXY; and 49,XXXXY, respectively. Error bars are presented as mean ± STD

To assess whether the significant *MAOA* methylation differences in Fig. 1d between neurotypical 46,XY and 49,XXXXY individuals were dependent on the number of X chromosomes, we compared CpG methylation at the *MAOA* first intron in males with different numbers of X chromosomes. We also chose another region within *AR* whose CpG methylation levels have been linked to gene regulation [31, 32]. Similar to individuals with 49,XXXXY, methylation levels for individuals with 47,XXY and 48,XXXY (for *MAOA* only) at both regions were higher at each CpG compared to the neurotypical 46,XY males in a copy number-dependent manner. For instance, CpG-1 in *MAOA* showed a copy number-dependent increase in DNA methylation from 10.4% in 46,XY to 54.3% in 49,XXXXY samples (Fig. 2a). The relationship between X chromosome number and DNA methylation was significant (*R*^2^ = 0.87, *P* = 6.8 × 10^−27^, Fig. 2b). Similar results were obtained for other *MAOA* CpGs tested. Similar methylation patterns (Fig. 3a) and statistical significance (*R*^2^ = 0.85, *P* = 9.4 × 10^−9^, Fig. 3b) were observed for CpG-1 of the *AR* locus. For the *AR* analysis, the samples also included DNA collected from the mothers of probands. These parental samples were included as 46,XX to compare against the 47,XXY samples, and similar DNA methylation levels were observed in both groups (Fig. 3a). We also sought to compare observed methylation values at *MAOA* and *AR* with hypothetical methylation values for the active and the inactive X chromosomes derived from 46XY, 46,XX, and 47,XXY samples. For the majority of the CpGs in both *MAOA* and *AR*, DNA methylation levels in 47,XXY and 49,XXXXY groups for both genes and 48,XXXY for *MAOA* were consistent with there being only one active X chromosome. For example, *MAOA* CpG-2 in the 46,XY was 6.9%, and this number was used to assign a value of 6.9% to the active X chromosome (Xa) in the 47,XXY group. An assumption was made that the second X chromosome in the 47,XXY sample was inactive (Xi) with an estimated 98.1% methylation [(98.1% + 6.9%)/2 = 52.5%] based on the experimentally obtained CpG-2 methylation (52.5%) in the 47,XXY samples. These derived values for Xa and Xi for CpG-2 were then used to assign hypothetical values to each X chromosome in the 48,XXXY and 49,XXXXY groups to assess whether the observed methylation levels corresponded to specific numbers of Xa and Xi in each group. For CpG-2, the estimated methylation values for 47,XaXiXiY and 49, XaXiXiXiY were 67.7% and 75.3%, respectively. Notably, these estimated values were similar to the observed pyrosequencing values at CpG-2 for 47,XXXY and 49,XXXXY groups at 67.5% and 70.8%, respectively. However, some CpGs showed methylation levels that were consistent with a scenario where all of the X chromosomes have been inactivated, i.e., 47,XiXiXiY or 49,XiXiXiXiY. For instance, the observed methylation levels at CpG-1 of *MAOA* in 49,XXXXY was 54.3%, which showed no statistically significant deviation from the 56.4% methylation predicted from all four X chromosomes being inactive (Table 3). *P *values for the differences between the hypothetical combinations of Xa/Xi and observed methylation levels are provided in Additional file 3: Table 2.

**Table 3:** Predicted and observed methylation levels for XY, XXY, XXXY, and XXXXY at the MAOA and AR loci

	XY	XXY			XXXY*					XXXXY					
	Xa/Obs	Xa	Xi	Obs	XaXaXa	XaXaXi	XaXiXi	XiXiXi	Obs	XaXaXaXa	XaXaXaXi	XaXaXiXi	XaXiXiXi	XiXiXiXi	Obs
MAOA															
CpG-1	10.4	10.4	56.4	33.4	10.4	25.8	**41.1**	56.4	47.2	10.4	21.9	33.4	44.9	**56.4**	54.3
CpG-2	6.9	6.9	98.1	52.5	6.9	37.3	**67.7**	98.1	68.4	6.9	29.7	52.5	**75.3**	98.1	70.8
CpG-3	5.6	5.6	45.9	25.7	5.6	19.0	32.5	**45.9**	39.6	5.6	15.7	25.7	35.8	**45.9**	41.2
CpG-4	5.9	5.9	91.8	48.9	5.9	34.6	**63.2**	91.8	59.9	5.9	27.4	48.9	**70.3**	91.8	63.1
CpG-5	6.0	6.0	83.0	44.5	6.0	31.7	**57.3**	83.0	61	6.0	25.2	44.5	**63.7**	83.0	69.0
CpG-6	4.2	4.2	77.5	40.9	4.2	28.7	**53.1**	77.5	53.0	4.2	22.5	40.9	**59.2**	77.5	62.0
CpG-7	7.6	7.6	98.1	52.9	7.6	37.8	**67.9**	98.1	72.2	7.6	30.2	52.9	**75.5**	98.1	78.3
AR															
CpG-1	3.0	3.0	34.2	18.6	NA	NA	NA	NA	NA	3.0	10.8	18.6	26.4	**34.2**	31.0
CpG-2	4.6	4.6	37.1	20.8	NA	NA	NA	NA	NA	4.6	12.7	20.8	**28.9**	37.1	28.4
CpG-3	7.1	7.1	82.7	44.9	NA	NA	NA	NA	NA	7.1	26.0	44.9	**63.8**	82.7	54.9
CpG-4	10.7	10.7	38.6	24.7	NA	NA	NA	NA	NA	10.7	17.7	24.7	31.6	**38.6**	36.5

^*^Methylation levels for XXXY were not available (NA) for AR, as there were no XXY probands. Xa denotes active X chromosome, and Xi denotes inactive X chromosome. Numbers in bold denote the predicted methylation values of Xa and Xi combinations that gave the closest value to that observed (Obs) experimentally by pyrosequencing. For XY, Xa was based on observed methylation at the X chromosome. For XXY, Xi was calculated based on Xa values from XY. For XXXY and XXXXY, all of the Xa and Xi were predicted based on values from XY and XXY and then compared to actually observed values from XXXY and XXXXY by pyrosequencing. All observed methylation vales have been adjusted by effects of testosterone

### CpG methylation at autosomes

**Fig. 4:**
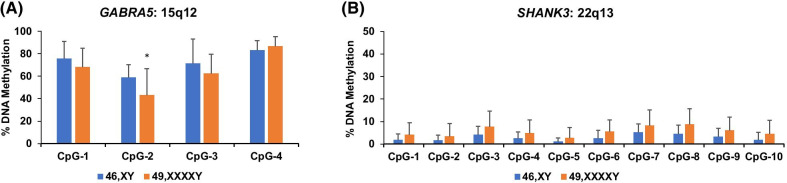
Methylation levels at candidate autosomal loci between 46,XY and 49,XXXXY. Genomic DNA extracted from saliva was used for bisulfite pyrosequencing of putative regulatory regions at two genes involved in neurodevelopment and neurotransmission: GABA type A receptor alpha5 subunit (*GABRA5*) on 15q12 (**a**) and SH3 multiple ankyrin repeat domains 3 (*SHANK3*) on 22q13 (**b**). Error bars are presented as mean ± STD. *P < 0.05

DNA methylation was also investigated at the GABA receptor 5 (*GABRA5*)/GABA receptor 3 (GABRB3) and *SHANK3* loci that are located on chromosomes 15q12 and 22q13, respectively. These loci were chosen based on their association with neurodevelopmental disorders such as autism [33–36] and served as negative controls to the significant across-group methylation differences observed on the X chromosome. There were no significant differences in CpG methylation between the 46,XY and 49,XXXXY groups at these two loci, although one CpG in *GABRA5* was slightly lower in methylation for 49,XXXXY (CpG-2, *P* = 0.04, Fig. 4a, b).

### Association between methylation and behavioral assessment

To assess whether behavioral differences between individuals with 49,XXXXY and neurotypical 46,XY were associated with methylation, BRIEF and CBCL scores were compared against *MAOA* and *AR* CpG methylation levels. The greatest association was observed with the CBCL externalizing behavior, where a 10% increase in methylation at *MAOA* CpG-2, CpG-3, CpG-4, and CpG-7 was significantly associated with at least a 2.5-point increase on the externalizing behavior scale (Table 4). The associations with the CBCL internalizing subscale were not significant, and only marginal significance was achieved for CpG-1 (*P* = 0.08), CpG-2 (*P* = 0.06), and CpG-7 (*P* = 0.08) on the CBCL total scale. There were no statistically significant associations between *MAOA* methylation and any of the BRIEF subscales. For the CpGs in *AR*, only CpG-1 was significantly associated (*P* = 0.03) with the BRIEF Behavioral Regulation Index, with a 10% increase in methylation correlating to a 15.9-point (95% CI: 6.3, 25.4 points) increase in this scale. Similar to the *MAOA* CpG sites, there was only marginal significance with the CBCL total scale for CpG-1 (estimated 9.9-point increase, *P* = 0.07) and CpG-2 (estimated 13.3-point increase, *P* = 0.09).

**Table 4:** Association between DNA methylation and behavior reports in individuals with 49,XXXXY

	CBCL total problems (95% CI)	*P *value	CBCL externalizing problems (95% CI)	*P *value	CBCL internalizing problems (95% CI)	*P *value
MAOA CpG-1	0.32 (− 0.03, 0.67)	0.08	0.30 (− 0.03, 0.62)	0.08	0.32 (− 0.09, 0.73)	0.13
MAOA CpG-2	0.27 (0.0002, 0.54)	0.06	0.31 (0.07, 0.55)	0.02	0.16 (− 0.16, 0.49)	0.33
MAOA CpG-3	0.33 (− 0.07, 0.73)	0.12	0.40 (0.04, 0.76)	0.04	0.23 (− 0.24, 0.70)	0.35
MAOA CpG-4	0.25 (− 0.06, 0.56)	0.13	0.32 (0.04, 0.60)	0.03	0.12 (− 0.25, 0.50)	0.52
MAOA CpG-5	0.20 (− 0.07, 0.47)	0.17	0.24 (− 0.004, 0.49)	0.06	0.15 (− 0.17, 0.47)	0.38
MAOA CpG-6	0.22 (− 0.07, 0.50)	0.15	0.26 (− 0.001, 0.52)	0.06	0.15 (− 0.18, 0.49)	0.38
MAOA CpG-7	0.22 (− 0.02, 0.45)	0.08	0.27 (0.06, 0.47)	0.02	0.13 (− 0.15, 0.40)	0.38
AR CpG-1	0.99 (− 0.14, 1.84)	0.07	1.05 (− 0.002, 2.11)	0.11	0.56 (− 0.28, 1.41)	0.25
AR CpG-2	1.33 (− 0.10, 2.57)	0.09	1.38 (− 0.16, 2.92)	0.14	0.71 (− 0.51, 1.93)	0.31
AR CpG-3	0.36 (− 0.44, 1.16)	0.42	0.32 (− 0.63, 1.26)	0.54	0.19 (− 0.48, 0.86)	0.61
AR CpG-4	1.16 (− 0.46, 2.78)	0.22	1.13 (− 0.84, 3.09)	0.31	0.68 (− 0.74, 2.10)	0.39

	BRIEF Behavioral Regulation Index (95% CI)	*P *value	BRIEF Metacognition Index (95% CI)	*P *value	BRIEF Global Executive Composite (95% CI)	*P *value
MAOA CpG-1	0.23 (− 0.23, 0.68)	0.33	0.05 (− 0.32, 0.42)	0.80	0.10 (− 0.30, 0.50)	0.63
MAOA CpG-2	0.23 (− 0.14, 0.60)	0.24	0.03 (− 0.28, 0.33)	0.86	0.09 (− 0.24, 0.41)	0.60
MAOA CpG-3	0.25 (− 0.30, 0.79)	0.39	0.06 (− 0.39, 0.50)	0.81	0.11 (− 0.36, 0.59)	0.65
MAOA CpG-4	0.31 (− 0.11, 0.74)	0.16	0.03 (− 0.33, 0.39)	0.87	0.12 (− 0.26, 0.50)	0.54
MAOA CpG-5	0.10 (− 0.26, 0.47)	0.58	− 0.02 (− 0.32, 0.27)	0.88	0.01 (− 0.31, 0.32)	0.97
MAOA CpG-6	0.21 (− 0.17, 0.59)	0.29	0.03 (− 0.28, 0.35)	0.85	0.08 (− 0.26, 0.42)	0.65
MAOA CpG-7	0.18 (− 0.14, 0.51)	0.28	0.02 (− 0.25, 0.28)	0.90	0.07 (− 0.22, 0.35)	0.66
AR CpG-1	1.59 (0.63, 2.54)	0.03	0.97 (0.14, 1.80)	0.08	1.11 (− 0.06, 2.28)	0.13
AR CpG-2	1.99 (0.41, 3.58)	0.07	1.22 (− 0.05, 2.48)	0.13	1.40 (− 0.32, 3.12)	0.19
AR CpG-3	0.69 (− 0.40, 1.79)	0.28	0.45 (− 0.33, 1.22)	0.32	0.42 (− 0.63, 1.47)	0.48
AR CpG-4	2.17 (0.26, 4.07)	0.09	1.55 (0.30, 2.80)	0.07	1.55 (− 0.44, 3.53)	0.20

The *P *value threshold for statistical significance after Bonferroni correction for the number of CpG sites tested was 0.005

### Impact of testosterone on behavior and methylation

Males with 49,XXXXY typically undergo testosterone treatment (*T*) to promote optimal sexual and neurocognitive development [37]. First, scores on the CBCL and BRIEF were compared between the 49,XXXXY no-*T* and 49,XXXXY *T* groups. The 49,XXXXY *T* group showed a lower average score on the CBCL Total and the Externalizing Problems subscale, but not with any of the three BRIEF subscales (Table 5). For instance, receiving testosterone was associated with, on average, an 8.4-point reduction in the CBCL Total Problems score (*P* = 0.04). History of testosterone treatment was then compared with *MAOA* methylation. When examining the effect of different testosterone doses on changes in methylation, there were two nominally significant.

**Table 5:** Effect of testosterone replacement therapy on behavior in individuals with 49,XXXXY

	Received *T*	Did not receive *T*	*P *value
CBCL total problems	58.66 (40.0, 82.0)	67.10 (49.0, 78.0)	0.04
CBCL externalizing problems	54.3 (34.0, 75.0)	62.5 (48.0, 79.0)	0.04
CBCL internalizing problems	57.6 (34.0, 92.0)	62.9 (45.0, 75.0)	0.24
BRIEF BRI	57.2 (39.0, 88.0)	60.9 (50.0, 87.0)	0.54
BRIEF MI	56.7 (35.0, 75.0)	64.9 (50.0, 79.0)	0.11
BRIEF global	58.3 (36.0, 78.0)	64.7 (51.0, 85.0)	0.24

*MAOA* CpG sites that increased in methylation following EHT (< 60 months of age). Specifically, CpGs 2 and 4 both showed an increase of 6.5% (*P* = 0.01) and 6.4% (*P* = 0.047) in methylation, respectively. In contrast, individuals undergoing hormonal treatment later in life (> 60 months of age) did not show any significant *MAOA* CpG methylation differences compared to those that did not receive treatment (Table 6). To compare the effect of testosterone administered at different age on DNA methylation, we also examined methylation changes using those who received EHT (< 60 months) as reference. Methylation levels in individuals with 49,XXXXY who received HBT showed significantly higher methylation levels at *MAOA* CpG-7 (5.76%, *P* = 0.02). Although there was a general trend of increasing *MAOA* DNA methylation in individuals receiving testosterone treatment after 6 years of age, none of the increases were significant with the exception of CpG-3 (10.05%, *P* = 0.04, Table 7). We noticed a greater increase in *AR* methylation compared to *MAOA* methylation in individuals receiving testosterone later in life, although there were no nominally significant differences in DNA methylation at the *AR* locus with the exception of CpG-4 in those receiving TRT after 11 years of age (28.37%, *P* = 0.05). Despite several promising CpGs that increase in DNA methylation with treatment, none of the *P *values for the HBT and TRT groups compared to the EHT group were statistically significant following correction for the multiple CpGs tested. When modeling the effect of testosterone and DNA methylation on CBCL or BRIEF outcomes, there was no statistically significant interactive effect between the two variables for any CpG sites.

**Table 6:** Effect of testosterone replacement therapy on DNA methylation in individuals with 49,XXXXY

MAOA	Did not receive *T*	Received < 60 (*N* = 11)		Booster 60–107 (*N* = 4)		Megabooster 108–131 (*N* = 3)		132 + (*N* = 3)	
		Methylation % change (95% CI)	*P *value	Methylation % change (95% CI)	*P *value	Methylation % change (95% CI)	*P *value	Methylation % change (95% CI)	*P *value
CpG-1	Ref	2.365 (− 1.15, 5.88)	0.20	− 1.95 (− 6.50, 2.60)	0.41	2.60 (− 3.49, 8.69)	0.41	− 0.53 (− 6.73, 5.66)	0.87
CpG-2	Ref	6.52 (1.59, 9.24)	**0.01**	1.07 (− 4.62, 6.77)	0.72	− 0.27 (− 7.92, 7.37)	0.95	6.93 (− 0.06, 13.91)	0.07
CpG-3	Ref	4.02 (− 0.36, 8.40)	0.09	− 4.44 (− 10.07, 1.19)	0.14	0.99 (− 6.99, 8.97)	0.81	1.67 (− 6.28, 9.63)	0.069
CpG-4	Ref	6.41 (0.50, 12.32)	**0.047**	1.83 (− 6.41, 10.07)	0.67	− 3.34 (− 14.32, 7.63)	0.56	6.45 (− 4.24, 17.15)	0.25
CpG-5	Ref	0.38 (− 1.57, 2.32)	0.71	0.13 (− 2.33, 2.59)	0.92	1.75 (− 1.45, 4.94)	0.30	− 0.42 (− 3.70, 2.86)	0.81
CpG-6	Ref	2.11 (− 2.24, 6.46)	0.35	− 2.00 (− 7.55, 3.53)	0.49	− 0.79 (− 8.29, 6.71)	0.84	5.10 (− 2.06, 12.25)	0.18
CpG-7	Ref	− 0.95 (− 4.18, 2.28)	0.57	2.74 (− 1.17, 6.66)	0.19	1.14 (− 4.33, 6.61)	0.69	1.93 (− 3.50, 7.35)	0.50

Ref = reference point for *T* treatment. The numbers in the top row denote age in months of *T* treatment. The *P *value threshold for statistical significance after Bonferroni correction for the number of CpG sites tested was 0.005

**Table 7:** Timing of testosterone replacement therapy on DNA methylation in individuals with 49,XXXXY

MAOA	Received < 60 (*N* = 11)	Booster 60–107 (*N* = 4)		Megabooster 108–131 (*N* = 3)		132 + (*N* = 3)	
		% Methylation change (95% CI)	*P *value	% Methylation change (95% CI)	*P *value	% Methylation change (95% CI)	*P *value
CpG-1	Ref	0.37 (− 6.58, 7.32)	0.92	4.04 (− 2.45, 10.53)	0.25	− 6.13 (− 2.45, 14.70)	0.19
CpG-2	Ref	− 1.17 (− 7.11, 4.77)	0.71	2.49 (− 3.29, 8.27)	0.42	5.82 (− 1.39, 13.04)	0.15
CpG-3	Ref	− 1.83 (− 9.38, 5.72)	0.64	3.14 (− 4.24, 10.52)	0.42	10.05 (1.85, 18.24)	**0.04**
CpG-4	Ref	− 0.9 (− 6.89, 6.71)	0.98	− 0.33 (− 7.13, 6.46)	0.93	4.38 (− 4.38, 13.13)	0.35
CpG-5	Ref	1.56 (− 2.16, 5.27)	0.43	2.09 (− 1.52, 5.71)	0.28	4.06 (− 0.47, 8.58)	0.11
CpG-6	Ref	− 3.88 (− 13.61, 5.84)	0.45	0.23 (− 9.79, 10.25)	0.97	5.61 (− 7.45, 18.66)	0.42
CpG-7	Ref	5.76 (1.61, 9.91)	**0.02**	0.75 (− 4.71, 6.20)	0.79	0.73 (− 6.65, 8.10)	0.85
AR							
CpG-1	Ref	6.46 (− 5.49, 18.42)	0.31	− 4.61 (− 16.80, 7.78)	0.49	− 2.18 (− 19.12, 14.77)	0.81
CpG-2	Ref	3.97 (− 14.25, 22.19)	0.69	9.43 (− 8.01, 26.86)	0.31	17.53 (− 4.76, 39.81)	0.15
CpG-3	Ref	− 0.34 (− 15.22, 14.53)	0.97	8.39 (− 5.55, 22.32)	0.27	16.43 (− 0.84, 33.71)	0.09
CpG-4	Ref	4.26 (− 18.00, 26.52)	0.72	12.41 (− 8.65, 33.47)	0.28	28.37 (3.78, 52.96)	**0.05**

Ref = reference point for *T* treatment. The numbers in the top row denote age in months of *T* treatment

^1^Confidence intervals were estimated using the likelihood ratio, while *P *values were calculated using the Wald test. As a result, due to our small sample size and the estimated effect being very close to the 0.05 threshold, at times the confidence interval does not reject the null hypothesis. The *P *value threshold for statistical significance after Bonferroni correction for the number of CpG sites tested was 0.005

## Discussion

The objective of this pilot study was to assess the relationship among the number of supernumerary X chromosomes, CpG methylation at different loci on the X chromosome, and parent report of behavior in males. In two parental questionnaires, the BRIEF and CBCL, significant differences were observed in both internalizing and externalizing behaviors between the 49,XXXXY group and both the 47,XXY and neurotypical 46,XY groups, which is consistent with a previous study by our group [15]. However, there were no significant differences between the 47,XXY and the 46,XY groups on the BRIEF and CBCL, which is in contrast to another study [11, 38]. The extra three X chromosomes in 49,XXXXY were known to cause greater effects on gene expression and behavior than the one extra X chromosome in 47,XXY, as supported by our study.

For males with supernumerary X chromosomes, increased gene expression could be a result of incomplete inactivation of the X chromosomes. Elevated expression of genes on the X chromosome due to duplication at different X-linked loci is associated with neurodevelopmental disorders. For example, duplications of Xq28 that include the *MeCP2* gene have been described in male patients that present with severe developmental delay and neurological effects [39]. Another duplication, at the Xq12-q13.3 position, was associated with developmental delay, autistic features, and increased dosage of genes in the duplicated region [40]. We studied X chromosome inactivation indirectly by measuring DNA methylation at different locations. Considering that the inactive X chromosome has much higher levels of methylation than the active X chromosome, each additional inactive X chromosome is expected to increase the observed methylation at each gene.

Due to its involvement in the oxidative deamination of monoamines such as dopamine, norepinephrine, and serotonin, *MAOA* is a noteworthy candidate to investigate the role of DNA methylation in behavior. It has been shown that genetic variants linked to low MAOA activity have been associated with increases in aggression and antisocial behavior in neurotypical 46, XY males exposed to childhood trauma [41, 42]. There is also genetic evidence for *MAOA* in attention deficit hyperactivity disorder (ADHD) [43], a disorder observed in individuals with 47,XXY [44]. In fact, studies have shown that *MAOA* may be one of the genes targeted by methylphenidate or Ritalin [45]. Other studies found *MAOA* hypomethylation in female patients with panic disorder when compared to neurotypical counterparts [46, 47]. In the current study, CpG methylation was much higher in the *MAOA* intronic region in the 49,XXXXY group compared to the 46,XY controls. This finding was expected due to the extra number of X chromosomes that are presumed to have been inactivated, in part, by DNA methylation. However, we found evidence of multiple loci where methylation levels were different from expected. For instance, several intronic CpGs in *MAOA* did not display the expected greater methylation levels in the 49,XXXXY group compared to the 46,XY controls, whereas others reflected methylation levels that were consistent with all four of the X chromosomes being methylated and inactive. A study investigating the same intronic CpGs as ours reported a significant inverse correlation between methylation levels and MAOA enzymatic activity in the brain [30]. If these CpGs can mediate transcription, then increase in methylation of these CpGs would decrease *MAOA* gene activity, consistent with the above studies that examined low MAOA activity, aggression, and antisocial behavior. Studies of additional regulatory regions as well as gene expression are needed to delineate the functional consequences of DNA methylation in *MAOA*.

Methylation levels at *AR* were also investigated in our study due to its reported influence on male behavior. The androgen receptor is necessary for mediating androgen signaling to shape the male sexual phenotype. Polymorphisms in the androgen receptor gene have also been associated with aggression [48, 49]. Similar to the *MAOA* locus, methylation levels at one region in *AR* displayed the expected increased levels of methylation in boys with extra X chromosomes but another region did not. The less-than-expected methylation levels evident at some loci of the *AR* gene may be associated with the upregulation of *AR* expression as a compensatory response to hypogonadism.

The *MeCP2* gene encodes a protein that regulates gene expression and maintains normal CNS functioning by binding to methylated CpGs [50]. Mutations in *MeCP2* causes Rett syndrome, which is characterized by impairments in language, loss of motor coordination, and severe autistic features [51]. A recent study reported that the inactivation of the *MeCP2* gene was also associated with an increase in anxiety-driven behavior in males [52]. The *MeCP2* locus also did not display drastically elevated methylation levels in the 49,XXXXY group. However, only two CpGs in an intronic GRE were tested to demonstrate that not all X-linked regions show X chromosome number-dependent increase in DNA methylation. A thorough investigation of additional CpGs is needed.

The effect of testosterone treatment on behavior and DNA methylation was also assessed. Receiving testosterone was associated with lower average CBCL total scores and CBCL externalizing subscales, suggesting that treatment may have a positive effect on mental health. The beneficial effects associated with testosterone and behavior have been reported for more than a decade. Notably, testosterone treatment regulates both *MAOA* and *AR* expression [53–55] with one study reporting an interaction between testosterone treatment and an allelic variant in MAOA [56]. Generally, DNA methylation seems to be responsive to hormone treatment. In individuals with gender dysphoria who received cross-sex hormone therapy (CHT), those who underwent 12 months of estrogenic treatment to transition from male to female underwent an increase in methylation of the *AR* gene. Individuals who transitioned from female to male and underwent 12 months of testosterone treatment showed an increase in estrogen receptor alpha (*ESR1*) methylation [57]. Our findings suggest that hormone treatment can impact epigenetic mechanisms, although it is unclear whether this is a causal relationship. Our findings in this pilot study that testosterone treatment was associated with an increase in DNA methylation in several CpGs in *MAOA* and *AR* are preliminary and warrant further interrogation in a larger study of boys with 47,XXY as well as 48,XXXY and 49,XXXXY.

Several notable studies have investigated DNA methylation levels in individuals with Klinefelter syndrome [20, 58–60]. These studies were genome-wide in design and have identified hundreds of syndrome-relevant genes or regions that were differentially methylated between 47,XXY and their 46,XY counterpart. However, there are no studies that have examined DNA methylation in 49,XXXY, except for a study that investigated the FMR1 CGG repeat methylation in the context of a screening tool for newborns [61]. To date, our study is one of the first to focus on DNA methylation and have attempted to link methylation with behavior and androgen treatment in individuals with 49,XXXXY.

The current study has several limitations. First, although DNA methylation can provide a mechanistic understanding of how likely a gene is to be expressed, it is unclear whether any of the specific CpG methylation differences observed among the groups may account for differential gene expression. However, we state that the methylation levels, especially at the *MAOA* locus, have been linked with MAOA activity, suggesting that they may play a functional role in the transcriptional activity of *MAOA*. Second, we cannot conclude from statistical association that *MAOA* or *AR* methylation is responsible for the behavioral deficits observed in the 49,XXXXY boys, although it is intriguing to consider. On the X chromosome alone, there are a myriad of genes that can affect behavior, and there are numerous CpGs that may potentially regulate the function of any single gene. Given the relatively small number of CpGs tested in this study, it is probable that other loci may play a role on behavior as well, which will be investigated in future studies. An animal model such as one that employs the disruption of MAOA activity may contribute to augmenting our understanding of these very important interactions. Third, another limitation stems from the subjective BRIEF and CBCL scores provided by the participants’ parents. More objective assessments, such as those administered for neurocognitive evaluations, are needed to better understand correlative relationships between behavioral scores and biological variables such as DNA methylation. We estimated the values of Xa and Xi based on experimental data from 46,XY and 47,XXY with the assumption that the Xa and Xi had the identical methylation levels across the different groups. It may be the case that both active and inactive X chromosomes harbor variable methylation levels in the different groups tested. Such a finding would undermine our assumption. However, our results show that methylation levels of many of the CpGs obey our assumption. Given the large number of neurodevelopmental genes that reside on the X chromosome and their potential to affect the transcription of genes across the genome, it is likely that autosomal CpGs undergo epigenetic changes with extra number of X chromosomes. A comprehensive genome-wide approach is needed and will be utilized in future investigations. We acknowledge the limitations associated with using saliva for molecular analysis, as it does not provide a sterile medium for reliably extracting full-length mRNA [62]. This meant that we could not easily assay for gene expression of *MAOA*, *AR*, or *MeCP2*, which is an endeavor much more easily accomplished with blood samples. Finally, we were unable to determine whether there was a statistical interaction between testosterone and *MAOA* methylation on the behavioral outcomes, as we were underpowered due to low sample size. However, these limitations provide fertile ground for future investigations into the intriguing relationship between genes, hormones, and behavior in the supernumerary X disorders.

## Conclusions

Our study provides preliminary evidence indicating that boys with multiple supernumerary X chromosomes and their resulting behavioral issues likely involve some genes on the X chromosome that are aberrantly methylated due to incomplete X chromosome inactivation. These findings warrant a more comprehensive and detailed investigation to understand the relationship between specific genes, biological underpinnings of the testosterone deficiency, the inactivation of X chromosomes, and the behavioral presentations.

## Materials and methods

### Participants

In this study, there were 29 boys with 47,XXY and 27 boys with 49,XXXXY who were referred by their physicians or ancillary healthcare providers for comprehensive neurodevelopmental evaluations. Inclusion for this study required confirmation of 47,XXY or 49,XXXXY diagnosis via karyotype. Three individuals with three X chromosomes (two with XXXY and one with XXXYY) were also included only for the epigenetic analysis at *MAOA*. Males who were found to be born premature (i.e., ≥ 37 weeks), have mosaicism, copy-number variants, and/or other co-existing genetic disorders were excluded from the study. Participants were thoroughly evaluated by their pediatric endocrinologist including necessary laboratory tests and physical examination. The endocrinologist then administered HRT to the participant on an individual basis. Fourteen neurotypical boys (46, XY) were also included in this study to serve as controls. Not all participants submitted saliva samples for DNA methylation analysis.

### Behavioral report measures

The school-aged version of the Child Behavior Checklist (CBCL) [23] is a behavioral assessment for children ranging between 6 and 18 years of age. This form contains 118 questions in which a parent rates the child’s behavior over the past two months using a 3-point scale (1 = not true, 2 = somewhat true, 3 = very true). The CBCL measures a child’s expression of internalizing problems (anxious, withdrawn, depressed), externalizing problems (aggression, rule-breaking behavior) and other problems (attention, social problems, thought problems) in terms of a raw score. This raw score is then converted to a standardized percentile in which scores at the 50th percentile are considered average. Any score below the 93rd percentile is considered normal, scores between the 93rd and 97th percentiles are borderline, and any score above the 97th percentile is clinical. Borderline and clinical scores imply significant behavioral deviation compared to a normative population.

The school-aged edition of the Behavior Rating Inventory of Executive Function-2 (BRIEF-2) [24] is an 86 question-long form designed to assess the behavior of children between ages 5 and 18. This form is intended to be completed by both parents and teachers. The BRIEF-2 uses three scales to measure behavioral regulation (inhibit, shift, and emotional control), and five scales to measure metacognition (initiate, working memory, plan/organize, organization of materials, and monitor). The global executive composite combines all these scales to arrive at a behavioral summary score. *T*-scores between 60 and 64 are considered slightly elevated, *T*-scores from 65 to 69 are considered at risk of clinical elevation, and *T*-scores above 70 are considered clinically elevated in terms of executive function.

### DNA extraction from saliva

Each of the participants provided ~ 1 mL of saliva sample in an Oragene Discover salivette tube (DNAGenotek, Ottawa, Ontario, Canada). DNA was extracted using the prepIT-L2P saliva DNA isolation kit according to the manufacturer’s instructions (DNAGenotek). Briefly, saliva samples were incubated at 50 °C for 1 h, after which the PT-L2P solution was added at 1/25 of the saliva volume, and samples were vortexed and incubated on ice for 10 min. After the centrifugation of impurities at 15,000 × g, the supernatant was mixed with 1.2 times the volume of 100% EtOH, and saliva DNA was precipitated at room temperature. Following centrifugation at 15,000 × *g*, the supernatant was discarded, and the DNA pellet was washed once with 70% EtOH. The dried DNA pellet was resuspended in 100 μL of TE buffer, and DNA quantity was obtained by a Qubit 2 Fluorometer (ThermoFisher Scientific, Waltham, MA). For each sample, 500 ng of DNA was used for subsequent bisulfite conversion, PCR amplification, and methylation analysis by pyrosequencing.

### Bisulfite pyrosequencing assays for DNA methylation

DNA (500 ng) was first bisulfite-converted using the EZ DNA Methylation-Gold Kit, according to the manufacturer's protocol (Zymo Research, Irvine, CA). Primers were designed against regions as described in Additional file 2: Table 1. Whenever possible, we included regions experimentally verified as binding sites for the glucocorticoid receptor, or glucocorticoid response elements (GREs) [25], since anxiety is a common feature in the SCA disorder [11, 15]. For each region, 2 sets of primers were designed. Thermocycling was performed using the Veriti thermal cycler (Life Technologies, Carlsbad, CA), and 25 ng of bisulfite-treated DNA was used with the first outer set of primers. An additional nested PCR was performed with 2 µL of the first PCR reaction and one biotinylated primer (other primer being unmodified). Amplification for both PCR steps consisted of 40 cycles (94ºC for 1 min, 53ºC for 30 s, 72 °C for 1 min). PCR products were confirmed on agarose gels. Pyro Gold reagents were used to prepare samples for pyrosequencing according to manufacturer's instructions (Qiagen, Germantown, MD). For each sample, biotinylated PCR product was mixed with streptavidin-coated sepharose beads (GE Healthcare, Chicago, IL), binding buffer, and Milli-Q water, and shaken at room temperature. A vacuum preptool was used to isolate the sepharose bead-bound single-stranded PCR products. PCR products were then released into a PSQ HS 96-plate containing pyrosequencing primers in annealing buffer. Pyrosequencing reactions were performed on the PyroMark 96MD System according to the manufacturer’s protocol (Qiagen). CpG methylation quantification was performed with the Pyro Q-CpGt 1.0.9 software (Qiagen). An internal quality-control step was used to disqualify any assays that contained unconverted DNA. Percentage of methylation at each CpG as determined by pyrosequencing was compared among different groups.

### Statistical analysis

#### X chromosome copy number analysis

We estimated the expected level of DNA methylation at *MAOA* and *AR* CpG sites by using the observed methylation in XY and multiplying it by the potential number of inactivated X chromosomes: *m*_*j*_ = *n* * *m*_1_, where m is the estimated average level of methylation at the CpG site, *j* is the total number of X chromosomes in a given sample, *n* is the number of inactivated X chromosomes (max(*n*) = *j*), and *m*_1_ is the average level of methylation observed among samples from neurotypical 46,XY individuals. We then determined the predicted methylation levels from the number of inactive X chromosomes that were closest to those experimentally obtained by bisulfite pyrosequencing. The observed vs. expected values were tested using the Kolmogorov–Smirnov test, with lack of statistical significance indicating that the observed was similar to the expected values. Finally, observed values were also adjusted by testosterone treatment. Same conclusions were reached by both sets of values, and therefore, only the adjusted methylation values are reported.

#### Methylation correlation with behavior

We tested whether methylation was correlated with behavior across the CBCL: (1) total problems and (2) internalizing and (3) externalizing subscales using ordinary least-squares regression. This was also done across the BRIEF subscales: (1) the behavioral regulation index, (2) the metacognition index, and (3) the global executive functioning composite. These models were conducted without adjustment and with adjustment for aneuploidy status, with status in the model defined as having 2 X chromosomes (47,XXY) to having 4 (49,XXXXY) to assess whether this differential aneuploidy accounted for the relationship between differential methylation and behavior.

#### Testosterone treatment’s correlation with methylation

We assessed whether testosterone treatment had an impact on methylation, focusing on *MAOA* due to its prior association with behavior [26–28]. Seventeen individuals with 47,XXY received testosterone treatment (47,XXY *T* group), whereas three individuals with 47,XXY did not (47,XXY no-*T* group). Fifteen individuals with 49,XXXXY received testosterone treatment (49,XXXXY *T* group), and twelve individuals with 49,XXXXY did not (49,XXXXY no-*T* group). Individuals with no definitive treatment status were excluded from analysis. Testosterone treatment (*T*) includes early hormonal treatment (EHT), hormonal booster treatment (HBT), and testosterone replacement therapy (TRT). EHT is defined as three intramuscular injections of 25 mg testosterone enanthate, typically administered to boys between 4 and 60 months of age. HBT encompasses three intramuscular injections of 50 mg testosterone enanthate administered to boys between 5 and 8 years of age. TRT, beginning at puberty and continuing for life, involves either daily application of testosterone using Androgel or weekly or monthly injections.

#### Testosterone treatment’s correlation with behavior

We tested for an association between testosterone treatment and scores on the CBCL and BRIEF subscales using ordinary least squares regression. In a separate model, we also tested whether there was an interactive effect between receiving any *T* and differential methylation at the *MAOA* CpG sites.

#### Multiple testing correction

We used a Bonferroni correction for methylation analyses using the number of CpG sites to control for the Type I error rate. With an intended alpha threshold of 0.05, across 11 CpG sites, we used a corrected alpha threshold of 0.005. Given prior studies showing overlap in the psychodevelopmental constructs the BRIEF and CBCL measure [29], we considered statistical analyses with these outcomes as one experiment and did not apply a correction.

## Supplementary Information

**Additional file 1: Supplementary Table 1**. Primers used for bisulfite pyrosequencing.

**Additional file 2: Supplementary Table 2**. P-values of one-sample Wilcoxon Signed rank test (non-parametric test of means) between the predicted and observed methylation levels for 46,XY, 47,XXY, 48,XXXY, and 49,XXXXY at the MAOA locus.

**Additional file 3**. Behavioral and epigenetic database used for analysis.

## Data Availability

The datasets used and/or analyzed during the current study are available as Additional file 3: Behavioral and epigenetic databased used for analysis.

## Abbreviations

ADHD: Attention deficit hyperactivity disorder
AR: Androgen receptor
ASD: Autism spectrum disorder
BRIEF: Behavior Rating Inventory of Executive Function
CBCL: Child Behavior Checklist
CHT: Cross-sex hormone therapy
EHT: Early hormonal treatment
ESR1: Estrogen receptor alpha
FMR1: Fragile X mental retardation 1
FMRP: Fragile X mental retardation protein
GABRA5: GABA receptor subunit alpha 5
GABRB3: GABA receptor subunit beta 3
GRE: Glucocorticoid response element
HBT: Hormonal booster treatment
HRT: Hormonal replacement therapy
KS: Klinefelter syndrome
MAOA: Monoamine oxidase A
MeCP2: Methyl-CpG binding protein 2
SCA: Sex chromosome aneuploidy
SHANK3: SH3 multiple ankyrin repeat domain 3
TRT: Testosterone replacement treatment
