# Supplementary material for: DNA methylation and behavioral dysfunction in males with 47,XXY and 49,XXXXY: a pilot study
Source: Clin Epigenetics. 2021 Jul 1;13:136. doi: 10.1186/s13148-021-01123-4 (PMC8252231; doi:10.1186/s13148-021-01123-4)
Supplement: Supplementary file 2 — Additional file 2: Supplementary Table 2. P-values of one-sample Wilcoxon Signed rank test (non-parametric test of means) between the predicted and observed methylation levels for 46,XY, 47,XXY, 48,XXXY, and 49,XXXXY at the MAOA locus. [file 13148_2021_1123_MOESM2_ESM.docx]

Supplementary Table 1: Primers used for bisulfite pyrosequencing

| Gene Coordinates | PCR Primers | Pyrosequencing Primers |
| --- | --- | --- |
| Androgen Receptor GRE  Chr X: 66,832,170 – 66,832,515 | 5’-TGGTAAAGTTTGGGATTAAATTTAATAT  5’-TTTACTAAAACTTAATAACTTTTCTTA  5’-TTGTTTGGGATTTTAGTTAGAGTGATT  5’-*AATATTTCTCTTTTTACTATATACCCTTAA | 5’-GTTAGAGTGATTTGTTTTGAA  5’-TGTATATGTTGTTTTTT  5’-GAAGTTTTTTTTAATTTTTTTATTTTAAGA  5’-TTATTTAGTGAAATAAT |
| AR Repeat  Chr X: 66,765,075 – 66,765,099 | 5’-TAAGGGAAGTAGGTGGAAGATTTAGTT  5’-ACCACRACRACTCCAAACTCTAAAAC  5’-AAGTTTAAGGATGGAAGTGTAGTTA  5’-*CAACCTCTCTCCAAATAACACTCC | 5’-GAGTTTTTTAGAATTTGTTTTAGAG |
| GABRA5  Chr 15: 27,139,285 – 27,139,313 | 5’-GATAGATTTTAAGGATTTTTGTTTGAAG  5’-CTTAACATCATAAAACCAACTCTTACAAAC  5’-AGGAGTGTTAGGTYGAGGATTGGAG  5’-*CCATCTCACCCCAAAACCCACTAACT | 5’-GGGTAGAAGTTTTGTTTAGA |
| MAOA Intron 1  Chr X: 43,515,609 – 43,515,647 | 5’-GATTTAGGAGYGTGTTAGTTAAAGT  5’-TTATTATATCTACCTCCCCCAA  5’-AGTTAAAGTATGGAGAATTAAG  5’-*CACACCACCAACAAAACTAACAC | 5’-AAAGTATGGAGAATTAAGAGAAGG |
| MAOA GRE  Chr X: 43,567,173 – 43,567,369 | 5’-GTATAGGTATGTTTTTAAATTAATTTGGA  5’-ATCAAATTCATTTTATACCTACACTCTA  5’-GGTTATTTGGTAGTTTTTTTAGTGATTTA  5’-*ACTAATTTTCCAAAATAACCTTTAATCTCTA | 5’-AATATAAAAGTATAGAGAGATT  5’-AAAGTTATTTAAGTGTTTTGGGGTA |
| MeCP2 GRE  Chr X: 153,312,587 – 153,312,724 | 5’-GTTAGAAATAGAATATATATAGTTGA  5’-ACCTAAAAAATACTTTCTTTATCCTTA  5’-ATTAGATAAAGTGAATGTAGATG  5’-*CTATTAAACATTCTTTATCAAATTATCCTAA | 5’-AGATAAAAGTTAAATTAATT  5’-AGAGTTGATTTGAAATAGTA |
| SHANK3  Chr 22: 51,158,954 – 51,159,110 | 5’-TTAGTTTTAAGTTYGGTTTGGA  5’-AAACTACCCACRACCAAAAC  5’-GTTTTATGATTATTTTGTAGGAT  5’-TCCTCCACCTACAACTACTTCACCAA | 5’-ATGATTATTTTGTAGGATT  5’-GATAGTTTTTAYGTTAATTTGG |

Genomic coordinates are obtained from the UCSC Genome Browser human assembly: GRCh37/hg19. The PCR primers are arranged so that the top two rows for each genomic region represent the two outer primers, and the bottom two rows represent the nested primers. The asterisks on the bottom primers reflect biotinylation necessary for pyrosequencing.
