# Supplementary material for: DNA methylation and behavioral dysfunction in males with 47,XXY and 49,XXXXY: a pilot study
Source: Clin Epigenetics. 2021 Jul 1;13:136. doi: 10.1186/s13148-021-01123-4 (PMC8252231; doi:10.1186/s13148-021-01123-4)
Supplement: Supplementary file 3 — Additional file 3. Behavioral and epigenetic database used for analysis. [file 13148_2021_1123_MOESM3_ESM.docx]

**Supplementary Table 2. P-values of one-sample Wilcoxon Signed rank test (non-parametric test of means) between the predicted and observed methylation levels for XY, XXY, XXXY, and XXXXY at the MAOA locus.**

|  | XY | XXY | | | XXXY* | | | | | XXXXY | | | | | |
| --- | --- | --- | --- | --- | --- | --- | --- | --- | --- | --- | --- | --- | --- | --- | --- |
| MAOA |  | P-value of calculated compared to observed | | | P-value of calculated compared to observed | | | | | P-value of calculated compared to observed | | | | | |
|  | Xa/Obs | Xa | Xi | Obs | XaXaXa | XaXaXi | XaXiXi | XiXiXi | Obs | XaXaXaXa | XaXaXaXi | XaXaXiXi | XaXiXiXi | XiXiXiXi | Obs |
| CpG-1 | 10.4 | <10^-6^ | <10^-6^ | 33.4 |  |  |  |  | 47.0 | <10^-6^ | <10^-6^ | <10^-6^ | <10^-6^ | **0.07** | 54.3 |
| CpG-2 | 6.9 | <10^-4^ | <10^-4^ | 52.5 |  |  |  |  | 67.5 | <10^-6^ | <10^-6^ | <10^-6^ | **0.001** | <10^-6^ | 70.8 |
| CpG-3 | 5.6 | <10^-6^ | <10^-6^ | 25.7 |  |  |  |  | 41.4 | <10^-6^ | <10^-6^ | <10^-6^ | 0.0002 | **0.001** | 41.2 |
| CpG-4 | 5.9 | <10^-6^ | <10^-6^ | 48.9 |  |  |  |  | 59.5 | <10^-6^ | <10^-6^ | <10^-6^ | **0.0005** | <10^-6^ | 63.0 |
| CpG-5 | 6.0 | <10^-6^ | <10^-6^ | 44.5 |  |  |  |  | 59.7 | <10^-6^ | <10^-6^ | <10^-6^ | **0.37** | <10^-5^ | 69.0 |
| CpG-6 | 4.2 | <10^-6^ | <10^-6^ | 40.9 |  |  |  |  | 53.8 | <10^-4^ | <10^-4^ | <10^-4^ | **0.03** | <10^-4^ | 62.0 |
| CpG-7 | 7.6 | <10^-6^ | <10^-6^ | 52.9 |  |  |  |  | 71.2 | <10^-6^ | <10^-6^ | <10^-6^ | **0.002** | <10^-6^ | 78.3 |
|  |  |  |  |  |  |  |  |  |  |  |  |  |  |  |  |
| CpG-1 | 10.4 | 10.4* | 56.4* | 33.4 | 10.4 | 25.8 | **41.1** | 56.4 | 47.0 | 10.4 | 21.9 | 33.4 | 44.9 | **56.4** | 54.3 |
| CpG-2 | 6.9 | 6.9 | 98.1 | 52.5 | 6.9 | 37.3 | **67.7** | 98.1 | 67.5 | 6.9 | 29.7 | 52.5 | **75.3** | 98.1 | 70.8 |
| CpG-3 | 5.6 | 5.6 | 45.9 | 25.7 | 5.6 | 19.0 | 32.5 | **45.9** | 41.4 | 5.6 | 15.7 | 25.7 | 35.8 | **45.9** | 41.2 |
| CpG-4 | 5.9 | 5.9 | 91.8 | 48.9 | 5.9 | 34.6 | **63.2** | 91.8 | 59.5 | 5.9 | 27.4 | 48.9 | **70.3** | 91.8 | 63.0 |
| CpG-5 | 6.0 | 6.0 | 83.0 | 44.5 | 6.0 | 31.7 | **57.3** | 83.0 | 59.7 | 6.0 | 25.2 | 44.5 | **63.7** | 83.0 | 69.0 |
| CpG-6 | 4.2 | 4.2 | 77.5 | 40.9 | 4.2 | 28.7 | **53.1** | 77.5 | 53.8 | 4.2 | 22.5 | 40.9 | **59.2** | 77.5 | 62.0 |
| CpG-7 | 7.6 | 7.6 | 98.1 | 52.9 | 7.6 | 37.8 | **67.9** | 98.1 | 71.2 | 7.6 | 30.2 | 52.9 | **75.5** | 98.1 | 78.3 |

*Methylation levels for XXXY were not available (NA) for AR, as there were no XXY probands. Xa denotes active X chromosome, and Xi denotes inactive X chromosome. Information in the bottom half of the table is identical to that of Table 3 for MAOA and has been included as a reference. Numbers in bold denote the predicted methylation values of Xa and Xi combinations that gave the closest value to that observed (Obs) experimentally by pyrosequencing.

For XY, Xa was based on observed methylation at the X chromosome. For XXY, Xi was calculated based on Xa values from XY. For XXXY and XXXXY, all of the Xa and Xi were predicted based on values from XY and XXY and then compared to actually observed values from XXXY and XXXXY. The P-value threshold for statistical significance after Bonferroni correction for the number of CpG sites tested was 0.005.
